# Development of a production chain from vegetable biowaste to platform chemicals

**DOI:** 10.1186/s12934-018-0937-4

**Published:** 2018-06-13

**Authors:** Annemarie Schmidt, Gunnar Sturm, Christian Jonas Lapp, Daniel Siebert, Florencia Saravia, Harald Horn, Padma Priya Ravi, Andreas Lemmer, Johannes Gescher

**Affiliations:** 1 Department Applied Biology, Institute for Applied Biosciences, Karlsruhe Institute of Technology, Karlsruhe, Germany; 2 Institute of Microbiology and Biotechnology, University of Ulm, Ulm, Germany; 3 Chair of Water Chemistry and Water Technology, Karlsruhe Institute of Technology, Engler-Bunte-Institut, Karlsruhe, Germany; 4 State Institute of Agricultural Engineering and Bioenergy, University of Hohenheim, Stuttgart, Germany; 5 Institute for Biological Interfaces, Karlsruhe Institute of Technology, Karlsruhe, Germany

**Keywords:** Biowaste, Vegetable waste, Bioelectrochemical system, Acetoin, Propionate, Organic acids

## Abstract

**Background:**

A future bioeconomy relies on the development of technologies to convert waste into valuable compounds. We present here an attempt to design a biotechnological cascade for the conversion of vegetable waste into acetoin and electrical energy.

**Results:**

A vegetable waste dark fermentation effluent containing mainly acetate, butyrate and propionate was oxidized in a bioelectrochemical system. The achieved average current at a constant anode potential of 0 mV against standard hydrogen electrode was 177.5 ± 52.5 µA/cm^2^. During this step, acetate and butyrate were removed from the effluent while propionate was the major remaining component of the total organic carbon content comprising on average 75.6%. The key players with regard to carbon oxidation and electrode reduction were revealed using amplicon sequencing and metatranscriptomic analysis. Using nanofiltration, it was possible to concentrate the propionate in the effluent. The effluent was revealed to be a suitable medium for biotechnological production strains. As a proof of principle, the propionate in the effluent of the bioelectrochemical system was converted into the platform chemical acetoin with a carbon recovery of 86%.

**Conclusions:**

To the best of our knowledge this is the first report on a full biotechnological production chain leading from vegetable waste to the production of a single valuable platform chemical that integrates carbon elimination steps leading to the production of the valuable side product electrical energy.

**Electronic supplementary material:**

The online version of this article (10.1186/s12934-018-0937-4) contains supplementary material, which is available to authorized users.

## Background

In the last decades, the amount of food waste increased steadily. Globally, 1.3 billion tons of food are discarded per year. Hence, approximately one-third of all edibles is not used for consumption [1]. Globally, these organic residues are disposed to a large extend via landfills, thus leading to groundwater pollution, greenhouse gas emissions and spreading of pathogenic microorganisms [2, 3]. In Europe, biowaste is mostly degraded by composting and only minor shares are digested in biogas plants. Especially in composting facilities, a high amount of energy is lost in the form of heat and easily degradable carbon is lost in the form of carbon dioxide [4]. A favorable alternative to composting could be to use the organic waste streams for the production of bioenergy. This energy is preferably produced in the form of biogas. However, the quality of this biogas (55–65% methane content) does usually not comply with the regulations regarding the purity of natural gas (90–95% methane content) [5]. Hence, most often biogas is fed directly into a combined heat and power unit, since the purification of the gas is not economically viable for most of the biogas plant owners [6]. Moreover, methane is at least currently a rather cheap end-product and subventions are necessary to render the process economically viable for the producer. Another utilization of mixed biomass waste streams could be its direct application as substrate for the production of platform chemicals. Still, it is most likely the mixture of many different carbon sources in the waste streams that has so far hampered this approach [7, 8]. To solve this limitation, a fermentation routine consisting of a dark fermentation step followed by a bioelectrochemical oxidation is presented in this study that enables the stepwise conversion of biomass to a highly enriched single carbon source.

In a dark fermentation process, biomass is often fermented under slightly acidic and most often under high temperature conditions. Acidification leads to an inhibition of methanogenesis [9] and the degradation of organic matter stops at the acidogenesis step with the production of carbon dioxide, short- and middle-chain volatile fatty acids (VFAs) and hydrogen [10]. The main VFAs comprised in the final solution are acetic, propionic, butyric and valeric acid [10, 11]. Usually, the effluent of dark fermentation plants is neutralized and thereafter used for biogas production, or the main aim of the process is the production of hydrogen [12–15]. Generally, the profit from biomass based hydrogen production increases if the remaining constituents of the dark fermentation percolate are used as substrate for further processes [12, 13, 15–18].

The production of hydrogen is a consequence of prevailing acetate-butyrate fermentation, while higher propionate concentration in the fermentation vessel is associated with lower hydrogen yields. This is due to the overall stoichiometry of propionate fermentation that is accompanied by the consumption of electrons according to: glucose + 2 H_2_ → 2 propionate + 2 H_2_O [19].

Regarding the energy release under standard state conditions, propionic acid fermentation has higher ΔG^0^′ values compared to typical other fermentation processes (e.g. butyrate fermentation with − 247 kJ per mole glucose versus − 279.4 kJ per mole glucose for propionate fermentation). Hence, if hydrogen is available, organisms thriving via propionic acid fermentation could have a selective advantage and the concentration of propionic acid should increase.

Interestingly, propionate seems to be a rather recalcitrant substance under anoxic conditions. This might be due to the oxidation of succinate to fumarate in the tricarboxylic acid (TCA) cycle which is part of the propionate metabolism and commonly ubiquinol dependent. This ubiquinol dependence necessitates the presence of electron acceptors with rather high redox potentials. Furthermore, the toxicity of intermediates of propionic acid production seems to have selected for regulatory routines that prohibit propionate oxidation in some organisms even in the presence of alternative electron acceptors [20]. Certainly, there are organisms that consume propionate as carbon and electron source under anoxic conditions, but the mechanisms used by other organisms to strictly prevent anaerobic propionate consumption suggest that special adaptations are needed for its efficient anaerobic consumption.

Previous studies revealed that dark fermentation effluents can be used as substrate for bioelectrochemical systems [15–17, 21]. In these systems microorganisms couple the oxidation of an electron donor to the transfer of respiratory electrons to an anode surface [22]. Hence, the oxidation of organic compounds is directly coupled to the production of an electrical current [22, 23].

In this study, we report on the selective oxidation of acetate and butyrate from dark fermentation effluents in bioelectrochemical systems. Via amplicon sequencing and metatranscriptomics, we could identify potential biocatalysts for the oxidation of these substances on the anode surface. Different membrane systems were screened to concentrate the remaining propionate in the effluent. Finally, we show that this effluent is a proper carbon source for typical production organisms and present data for the aerobic biotechnological production of acetoin from biomass based propionate.

## Methods

### Chemicals

Chemicals and biochemicals were acquired from Sigma-Aldrich (Munich, Germany), Roth (Karlsruhe, Germany) and Promega (Mannheim, Germany). Enzymes were purchased from New England Biolabs (Frankfurt am Main, Germany).

### Bioelectrochemical system

The bioelectrochemical system (BES) used in this study was described by Sturm-Richter et al. [24]. The system is based on a two-chamber reactor of 270 ml volume including a three-electrode-setup. A carbon felt with 35 cm^2^ surface area served as working electrode, while a platinum net of 1.25 cm^2^ surface was used as the counter electrode. The reference of the system was a Ag/AgCl electrode. The electrodes in the two chambers were separated by a proton exchange membrane (Fumapem F-950, Fumatech, Germany). The working electrode was poised to 0 mV versus standard hydrogen electrode and the electrical current was measured by a potentiostat (uniscan instruments, PG8850RM). The electron acceptor of the cathode compartment was oxygen. The substrate of the system was changed under strictly anoxic conditions in an anoxic glove box. Coulombic efficiencies of the chronoamperometric experiments were calculated as described previously [25, 26].

### Substrate for the bioelectrochemical system

For all experiments, a slightly acidic hydrolysate was used as substrate (pH 5.5–6). The hydrolysate was produced from vegetable waste by dark fermentation at the University of Hohenheim [27] and was received either fresh or frozen. The hydrolysate was 100 µm filtered and contained mainly acetate (47.3–85.7 mM), butyrate (9.14–22.7 mM) and propionate (8.4–15.4 mM). The concentrations of the acids varied depending on the individual batch. The hydrolysate pH was adjusted to pH 7 with sodium hydroxide and it was purged with nitrogen gas to gain an anoxic substrate. The prepared hydrolysate was applied to the BES also under anoxic conditions. Over the whole experimental phase, the reactor was purged with a gas mixture of N_2_/CO_2_ (80%/20%). The pH was not further adjusted and samples were taken every 2–3 days.

### Bacterial strains and culturing conditions

A starter biofilm was developed on the anodes before the addition of the percolate to accelerate the initiation of the carbon oxidation process. The microorganisms used in this study are listed in Table 1 and were partly isolated from different waste water streams as ferric citrate reducing organisms (Epple et al. unpublished). All strains except *Geobacter sulfurreducens* were incubated in LB medium overnight. *Geobacter sulfurreducens* was pre-cultured for 2–3 days at 30 °C in a minimal medium according to Dolch et al. [28]. As electron donor, sodium acetate (10 mM) was added while 40 mM sodium fumarate served as electron acceptor.

**Table 1:** Bacterial strains and plasmids used in this study

	Genotype	Source
Strains for BES		
*Geobacter sulfurreducens barcode strain*	Synthetic sequence; 453226::kan barcode	[25]
*Shewanella oneidensis barcode strain*	Synthetic sequence; 71982::barcode	[25]
*Escherichia coli*		unpublished
*Enterococcus faecium*		unpublished
*Shewanella putrefaciens*		unpublished
Strains for production		
*E. coli K12 Δrnr* (JW5741-1)	F^−^, Δ*(araD*-*araB)567*, Δ *lacZ4787*(::rrnB-3), *λ*^−^, *rph*-*1*, Δ*(rhaD*-*rhaB)568*, Δ*rnr*-*729::kan*, *hsdR514*	[29]
*Corynebacterium glutamicum* (ATCC^®^ 13032™)		
Plasmids		
pMAL_*alsSD*	Amp^R^, P_lac_, *alsSD*	[30]

The barcode strains contain a short synthetic DNA sequence integrated in their genome that allows for the specific quantification of these organisms in mixed species communities [25]. The unpublished *E. coli*, *E. faecium* and *S. putrefaciens* strains were isolated previously from waste water as ferric iron reducing organisms (unpublished results)

Subsequently, all strains were used for inoculating the BES with a starting OD_600_ of 0.5. The strains were incubated in the system for 4 days in the above described medium without the addition of sodium fumarate, as the anode served as electron acceptor. After the pre-incubation, the medium was changed to the vegetable hydrolysate.

### Sample analysis and measurements

Samples were collected every 2–3 days for pH measurements as well as for quantifying the concentration of volatile fatty acids (VFAs) and total organic carbon (TOC). The amount of VFAs was determined via HPLC analysis (UltiMate3000; Thermo Scientific) using an Aminex HPX-87H column. Total organic carbon was measured via a Total organic carbon/Total nitrogen analyzer from Analytic Jena (multi N/C 2100). All samples were filtered through a 0.2 µm filter prior to analysis.

### Bioinformatic analysis

Genomic DNA as well as total RNA samples were obtained from anode biofilms using the Wizard Genomic DNA Purification Kit (Promega, Mannheim) and the RNA PowerSoil^®^ Total RNA Isolation Kit (MoBio/Qiagen, Hilden). 16S Illumina MiSeq sequencing (paired-end, 2 × 250 bp reads), rRNA depletion as well as Illumina RNA TruSeq sequencing (paired end, 2 × 150 bp) were conducted at IMGM Laboratories (Martinsried, Munich). The primers for the 16S Illumina MiSeq were Bakt_341F/Bakt_805R for bacteria and A519F/A906R for archaea (see Additional file 1: Table S1). 16S data analysis was conducted using the CLC Genomic workbench software (Qiagen, Hilden).

Metatranscriptome raw data was analysed using the software diamond v0.9.10.111 [31]. In total, 192 million reads were obtained from sequencing of eight lanes. Alignment of paired end Illumina reads was performed using the BLASTX algorithm with an e-value threshold of 10^−6^. 18 million reads matched with sequences of the NCBI nr-database. mRNAs corresponding to acetate kinase, phosphate acetyltransferase and acetyl-CoA synthetase encoding genes were used to assign transcriptomic data to putative acetate oxidizing microorganisms. Similarly, butyrate kinase and butyryl-CoA:acetate CoA-transferase were chosen for the metabolism of butyrate. Transcripts for *c*-type cytochromes were used to identify potential anode reducing microorganisms.

### Crossflow filtration and nanofiltration

After the BES step, the effluent was centrifuged (30 min up to 1 h, 30,000×*g*) and crossflow-filtered with a 0.2 µm or a 1 kDa crossflow module (mPES MidiKros^®^ Filter Modules D02-E20U-05-N or D02-E001-05-S in a KR2*i* TFF-System, SpectrumLabs, Breda, Netherlands). With the usage of the 1 kDa-Membrane instead of the 0.2 µm Membrane, parts of the dark brown colour could be filtered off in the crossflow system without affecting propionate concentrations. The subsequent nanofiltration was conducted under a nitrogen pressure of 4–4.5 bar, and with a nanofiltration membrane (Dow Filmtec, either NF90 or NF270, Sterlitech, USA) in a 350 ml stirring cell (Amicon, Merck Millipore, Germany), to achieve a concentration of propionate.

### Production of platform chemicals

The filtered and sterilized effluent from the BES was used as medium for an acetoin producing *Escherichia coli* strain as well as for *Corynebacterium glutamicum* (compare Table 1) to conduct simple growth experiments. HEPES (4-(2-hydroxyethyl)-1-piperazineethanesulfonic acid) was added to the fermentate to an end concentration of 50 mM to avoid pH fluctuation, and the pH was set to 7 before the fermentate was autoclaved.

*Corynebacterium glutamicum* and the *E. coli Δrnr* strain containing the pMAL_*alsSD* plasmid were subsequently used in cell suspension assays. It could already be shown before that another *E. coli* strain could produce acetoin with the here used pMAL_*alsSD* construct from glucose [30]. The production of acetoin, catalyzed by AlsS and AlsD, branches off at pyruvate, which is also the end-product of the 2-methylcitrate cycle used by *E. coli* to metabolize propionate.

*Corynebacterium glutamicum* is known for its biotechnological potential in amino acid production and could deliver interesting possibilities for the production of valuable chemicals from the here available propionate.

The cells were pre-incubated in LB-medium at 37 °C overnight and washed once before usage. Cells were added to the filtrated and sterilized fermentation broth to an OD_600_ of 2.5. Induction of the pMAL plasmid was achieved by addition of IPTG (50 µM). Induction of *C. glutamicum* for the release of glutamate to the cell medium was achieved by addition of Penicillin G (0.75 U/ml). Concentrations of acetoin in the liquid phase were measured using the Voges–Proskauer (VP) test according to Bursac et al. [32], while concentrations of glutamate were measured with the l-glutamic acid assay kit (Megazyme, Ireland).

## Results and discussion

### Current development from vegetable waste percolates

**Fig. 1:**
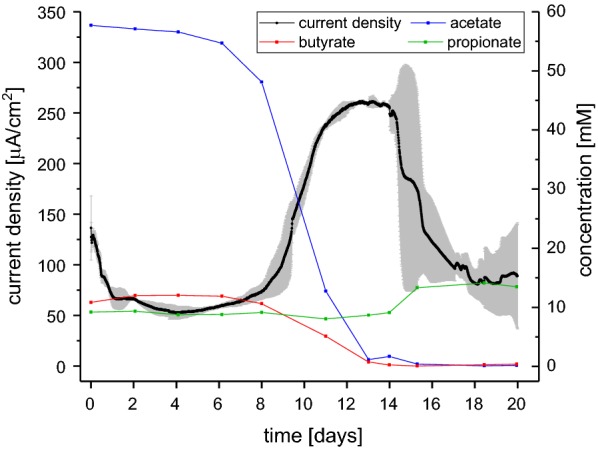
Representative graph of current density over time on the primary y-axis and concentrations of organic acids over time on the secondary y-axis. Current density is given in µA/cm^2^, concentrations in mM, while time is given in days. The grey area represents the standard deviation. This graph is representative for all 10 individual experimental phases

A vegetable fermentate originating from a biologically catalyzed acidic hydrolysis step was used as substrate for the BES without further filtration and after pH adjustment to pH 7. The BES were operated in batch mode and were pre-incubated with the laboratory model organisms *G. sulfurreducens* and *Shewanella oneidensis* as well as three isolates with 16S rRNA sequences that are most similar to *Shewanella putrefaciens*, *E. coli* and *Enterococcus faecium*. The preincubation was supposed to steer the oxidation process in the reactors towards anode reduction and to accelerate the anode reduction process. In previous experiments, it could be shown that, under certain process conditions, the preincubation can lead to stable biofilms that remain on the anode even under non-axenic conditions [25]. In Fig. 1, a representative experiment from the overall 10 bioelectrochemical experiments conducted in duplicate reactors was chosen to display the results of anode-assisted percolate oxidation. In other words, two independent reactors were fed 10 times each with percolate and we recorded the 20 experiment with regards to carbon consumption and current production. After a delay of 5 days, current density increased and reached its maximum value of 261.5 μA/cm^2^ between day 12 and 13. The length of the initial lag-phase decreased with the number of transfers to 1 or 2 days, indicating an adaptation of the anode community to the process of vegetable fermentate oxidation. The average maximum current density was 177.5 µA/cm^2^ (see Table 2). This value is comparable to BES that were operated with a mixture of boiled and raw potatoes [33, 34]. In the here presented experimental set conducted with dark fermentation percolates, the maximum in current could be correlated to the degradation of acetate and butyrate. In the beginning of the experiment, the dominant VFA was acetate, which accounted on average for 34% of the initial TOC, while butyrate and propionate comprised 19.4 and 9.6%, respectively. At the end of the bioelectrochemical batch conversions, acetate and butyrate were almost completely consumed, while the propionate content remained relatively stable or even increased. The depletion of the fermentate in acetate and butyrate went along with decreasing current densities, indicating that these acids were at least partly or indirectly consumed by anode respiring microorganisms. Overall, 79.3% of the initial TOC content were averagely eliminated (see Table 3). Since the general TOC content decreased and propionate was not consumed, its share of the overall TOC content (1.2 g/l) increased on average to 75.6%. Therefore, 25% of the residual TOC, corresponding to about 0.3 g/l, comprised a mixture of other compounds which corresponds to previous results [35]. Those are conceivably humic or fulvic acids, as the fermentate possesses a dark brownish color.

**Table 2:** Current density in µA/cm^2^ for all BES experiments

Experiment	Current density (µA/cm^2^)		
	Average	Maximum	Minimum
1	71.8 ± 39.5	189.7 ± 82.6	16.2 ± 1.6
2	119.9 ± 19.3	261.5 ± 0.8	50.0 ± 61.7
3	82.2 ± 50.1	158.5 ± 47.3	27.1 ± 35.7
4	94.8 ± 48.7	252.2 ± 86.0	53.7 ± 27.3
5	116.1 ± 76.0	151.2 ± 4.7	69.5 ± 71.0
6	80.2 ± 76.8	140.4 ± 86.5	27.5 ± 25.7
7	84.0 ± 40.9	106.1 ± 59.5	52.4 ± 2.3
8	117.5 ± 39.1	151.1 ± 4.7	50.9 ± 7.2
9	96.8 ± 22.1	225.7 ± 30.4	37.5 ± 3.5
10	106.1 ± 27.6	139.0 ± 34.7	59.3 ± 9.6
ø	96.9 ± 17.3	177.5 ± 52.5	44.4 ± 16.7

The same BES reactors were fed 10 times with fresh substrate and the current was measured via a potentiostat. From these measurements, the average, maximum and minimum values of current density were calculated

**Table 3:** Starting and end values of total organic carbon (TOC) for all individual experimental runs as well as the TOC elimination in g/h m^2^ and in %

Experiment	TOC *start* (g/l)	TOC *end* (g/l)	Decrease (g/h m^2^)	Decrease (%)
1	4.50 ± 0	0.67 ± 0.35	1.79	85.0
2	3.64 ± 0.18	0.39 ± 0.22	1.61	89.2
3	4.57 ± 0.28	0.72 ± 0.3	1.76	84.3
4	2.99 ± 0.04	0.67 ± 0.06	1.95	77.5
5	4.61 ± 0.20	0.75 ± 0.08	2.54	83.8
6	10.52 ± 0.27	6.16 ± 6.8	12.43	42.2
7	3.21 ± 0.07	0.55 ± 0.21	2.25	82.8
8	3.64 ± 0.02	0.30 ± 0.14	2.00	91.6
9	3.46 ± 0.04	0.39 ± 0.24	1.78	88.7
10	3.42 ± 0.11	1.1 ± 0.28	1.78	68.3
ø	4.46 ± 2.20	1.20 ± 1.76	2.99	79.3

The decrease in % is given for the whole experiment time

**Fig. 2:**
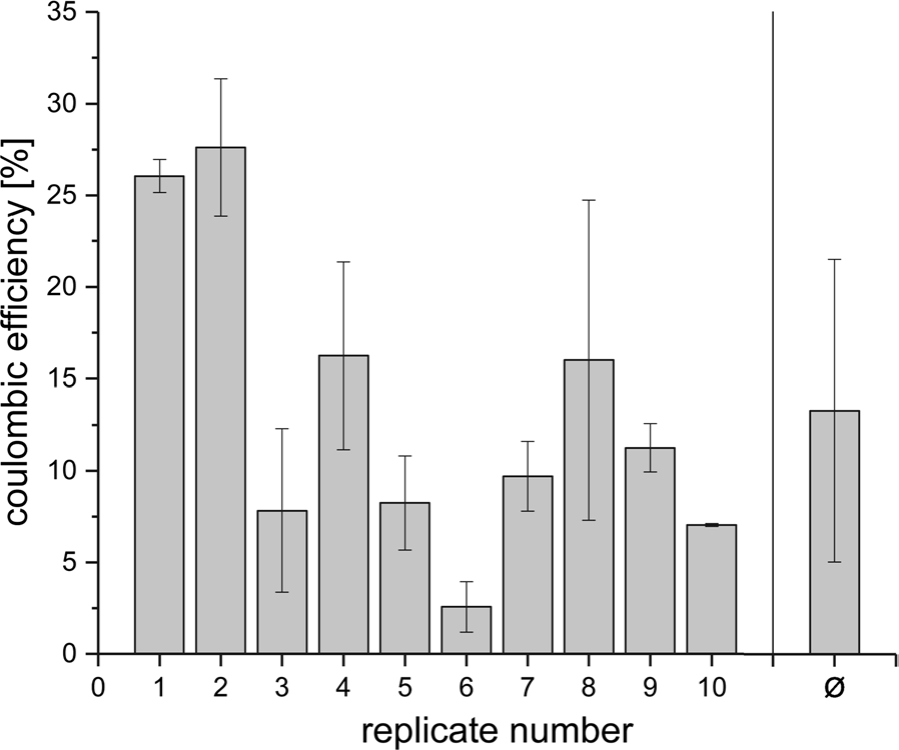
Calculated coulombic efficiency of the BES reactors over all individual experimental phases. The coulombic efficiency was calculated from the integral of the current curve and the quantity of electrons available from the amount of the degraded total organic carbon. In the last column, the average of all experimental phases is given

The efficiency of the organic carbon removal by anode respiration was quantified by calculating the coulombic efficiency of the process. The values for the individual experiments can be depicted from Fig. 2. The average value of 13.3% indicates the presence of competing processes like methanogenesis. Therefore, a 16S rRNA gene based phylogenetic as well as a metatranscriptomic analysis were conducted to investigate which processes might prevail on the anode surface and which organisms were the key biocatalysts of the biocenosis.

### 16S rRNA sequencing and bioinformatic analysis

**Fig. 3:**
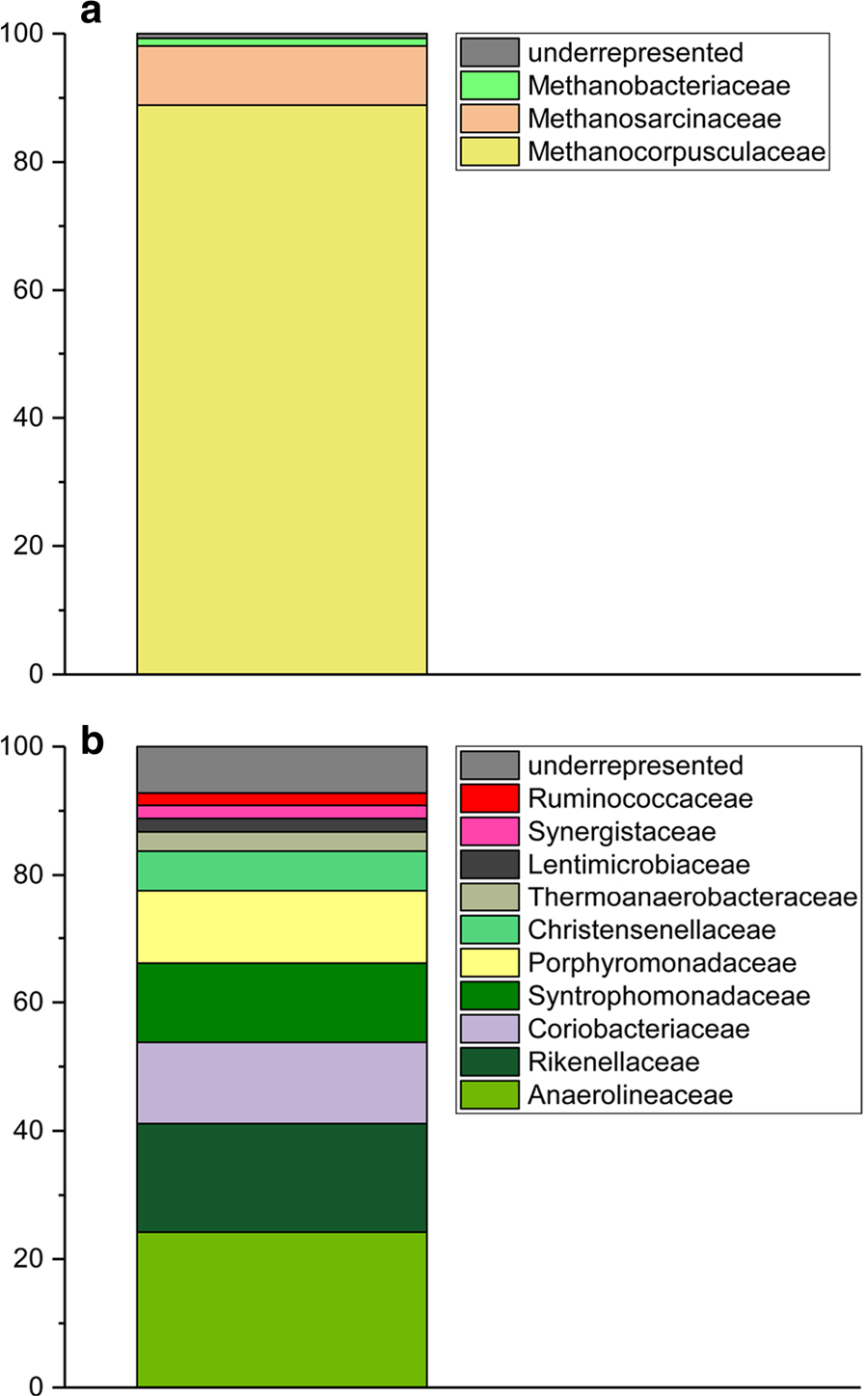
16S analysis. Samples were isolated from anodic biofilms of the BES reactors and used as template for a 16S Illumina MiSeq sequencing (paired-end, 2 × 250 bp reads). The primers for the 16S Illumina MiSeq were Bakt_341F/Bakt_805R for bacteria (**a**) and A519F/A906R for archaea (**b**) (see Additional file 1: Table S1)

The phylogenetic diversity of the community was assessed using sequencing of amplicons derived via archaea and bacteria specific primer pairs (see Additional file 1: Table S1). The distribution for archaea is depicted in Fig. 3a. The family *Methanocorpusculaceae* comprised 88.9% of the archaea in the community. Members of this family can utilize H_2_/CO_2_, formate, 2-propanol/CO_2_ and 2-butanol/CO_2_ as substrates for methane production. Growth on acetate could not be observed [36]. The *Methanosarcinaceae* were the second most abundant family (9.2%). Members of this family are known to be able to use a wide range of substrates, such as methylated amines, methanol, H_2_/CO_2_, acetate, dimethyl sulfide, methanethiol and carbon monoxide [37]. Hence, the low coulombic efficiency could be due to prevailing methanogenesis that could be sustained by hydrogen, CO_2_ and acetate producing primary and secondary fermentative organisms (see below).

The bacterial community (see Fig. 3b) was comprised mainly out of typical fermentative organisms, amongst others belonging to the *Chloroflexi* (24.2%), *Bacteroidetes* (31.3%) and *Clostridia* (25.6%), showing similarities to other anaerobic digester communities [38, 39]. Members of these phyla are capable of degrading cellulose. The *Anaerolineaceae* are the most abundant family in the here described community (24.2%) which correlates to other studies describing anaerobic digester or biogas communities [40–42]. Members of this family can hydrolyze cellulose and are frequently encountered in *n*-alkane degrading microbial communities [43]. Members of the Bacteroidetes are typically able to hydrolyze cellulose and to degrade proteins and amino acids to acetate and ammonia [44].

The frequency of detected 16S rRNA genes that are most similar to the *Coriobacteriaceae* (12.7%) was not expected. These organisms are gut bacteria, normally not belonging to typical anaerobic digester communities. They can thrive either via fermentation or anoxic respiration. Nevertheless, there is also evidence that organisms belonging to this family are electroactive, as they comprised a large fraction of a biocathode community. Moreover, the genome of the recently sequenced strain EMTCatB1 contains 18 putatively *c*-type cytochrome encoding genes, which could also be involved in the transfer of electrons onto anodes, possibly explaining the presence in this study [45].

We observed a rapid depletion of butyrate and acetate in our study. As already mentioned, methanogenesis could represent a process involved in the consumption of a portion of the available acetate. Additionally, methanogenesis could be involved in butyrate consumption if synthrophic organisms catalyze the intermediate step from butyrate to acetate and hydrogen. This oxidation of butyrate is known to be accomplished by acetogens of the genera *Syntrophus* and *Syntrophomonas* [46]. The family *Syntrophomonadaceae,* which includes both genera, comprised on average 12.4% of the bacterial 16S rRNA genes and is one of the main families of the bacterial community.

Although the anodes of the system were pre-incubated with *G. sulfurreducens*, a model organism for extracellular electron transfer, it was detectable in the 16S rRNA analysis by only 0.6%.

### Transcriptomic analysis

A transcriptomic analysis was used to assign acetate and butyrate degradation as well as electron transfer to microbial taxa within the anode biofilm. For acetate degradation, reads for acetate kinase/phosphate acetyltransferase as well as acetyl-CoA synthetase were assigned to archaeal as well as bacterial families.

**Fig. 4:**
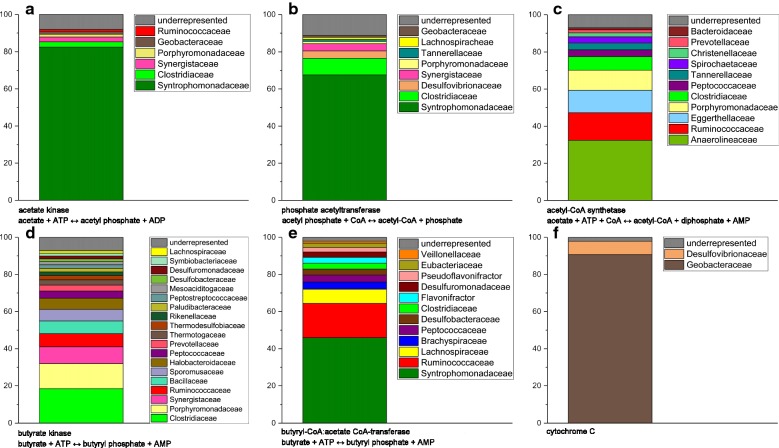
Transcriptomic analysis. Samples were isolated from anodic biofilms of the BES reactors and used as template for rRNA depletion as well as Illumina RNA TruSeq sequencing (paired end, 2 × 150 bp). Key genes of potential anaerobic metabolic pathways were phylogenetically binned: **a** acetate kinase, **b** phosphate acetyltransferase, **c** acetyl-CoA synthetase, **d** butyrate kinase, **e** butyryl-CoA:acetate CoA-transferase, **f** cytochrome C

Most of the reads for bacterial acetyl-CoA synthetase (see Fig. 4b) seem to be derived from members of the *Anaerolineaceae* (32.4%), which, as already described, are at least partly able to degrade cellulose and n-alkanes. They were further observed to produce acetate and provide it, for example, to acetoclastic methanogens [43], which can also be observed in the here studied community. In this case, acetyl-CoA synthetase would be responsible for acetate production and not for its degradation. Also, *Ruminococcaceae* (14.9%) were described as cellulolytic organisms, that produce acetate and formate or succinate [47, 48]. Moreover, also the *Porphyromonadaceae* (10.8%), the third most abundant group to which acetyl-CoA synthetase reads could be assigned, seems to contain primarily acetate producing organisms [49].

77.7% of the reads for acetate kinases could be assigned to members of the family *Methanosarcinaceae*, which is known to be acetoclastic. The other 22.3% for acetate kinases could be assigned to bacterial families (see Fig. 4a). A very similar distribution can be found for phosphate acetyltransferase (83.5% *Methanosarcinaceae*, 16.5% bacterial phyla), which catalyzes the step from acetate to acetyl phosphate. The latter can then be further processed to acetyl-CoA by acetate kinase. Also, the bacterial taxa assigned to produce acetate kinase and phosphate acetyltransferase are similar (see Fig. 4a, b).

The largest bacterial group to which acetate kinase reads could be assigned are the *Syntrophomonadaceae* with 81.7%, a family which could also be detected in the 16S analysis results. Although acetate kinase catalyzes a reversible reaction, organisms belonging to this family catalyze rather the production of acetate than its consumption [50].

Following, with 3.7 and 2.6% of bacterial acetate kinases respectively, are the *Clostridiaceae* and *Synergistaceae*. The latter could also be found in the 16S analysis, while *Clostridaceae* could not be detected (however, other families of the order *Clostridiales*).

Interestingly, 1.48% of the bacterial acetate kinase reads could be assigned to the family *Geobacteraceae*, a model family for exoelectrogenic organisms [51], which was also detected in the 16S analysis. Therefore, we can conclude that organisms of this family take part in acetate degradation. However, this family seems more important regarding the distribution of *c*-type cytochromes, proteins known to have an important function in extracellular electron transfer reactions [52] (Fig. 4f). Over 90% of the reads for *c*-type cytochromes could be designated to the family *Geobacteraceae*, indicating that organisms belonging to this family are involved in the extracellular electron transfer to the electrode. Of note, although the number of detected and assignable reads for bacterial acetate kinases and *c*-type cytochromes is rather similar (4337 versus 3797), members of the *Geobacteraceae* account for a 61-fold higher percentage of *c*-type cytochrome reads compared to acetate-kinase reads. Multiple c-type cytochromes are necessary for the transfer of electrons to the cell surface and this could be the reason for the observed acetate kinase to *c*-type cytochromes read-ratio. Nevertheless, it is also conceivable that acetate might not be the only electron donor used by members of the *Geobacteraceae*. Other potential electron sources could be hydrogen or direct interspecies electron transfer [53–55]. Still, in the latter case, *Geobacter* cells would most likely only operate as a cable to the anode and would not be able to use the redox potential difference between adjacent organisms and the anode for the production of cellular energy. Energy production would only be possible if the inward electron transfer pathway from the outer membrane through the periplasm and into the cytoplasm would be insulated from the outward electron transport chain to the cell surface.

Butyrate degradation seems to be conducted by syntrophic organisms of the family *Syntrophomonadaceae* and the order *Syntrophobacterales.* Butyrate degradation starts by activation of butyrate to butyryl-CoA, under usage of acetyl-CoA. This reaction is catalyzed by the butyryl-CoA:acetate CoA-transferase [56]. The read count for this enzyme is comparable to that of acetate kinase and c-type cytochromes, while reads for butyrate kinases, acetyl-CoA synthetases and phosphate butyryl transferases could be detected only in minor quantities. Therefore, we proposed for the here described BES, that the first step of acetate degradation is conducted by an acetate kinase rather than by acetyl-CoA synthetase and that the first step of butyrate degradation is catalyzed by a butyryl-CoA:acetate CoA-transferase rather than by a butyrate kinase.

The phylogenetic distribution of bacterial mRNAs for butyryl-CoA:acetate CoA-transferases is depicted in Fig. 4e. Similar to the results for acetate kinases, the major fraction of reads could be designated to members of the *Syntrophomonadaceae* (45.9%). With 18.5%, *Ruminococcaceae* represent the second largest group for this enzyme, and *Lachnospiraceae*, with 7.6%, the third most abundant group. The latter two families are known as butyrate-producing gut bacteria, often found in the human intestine [57–59].

In conclusion, a possible physiological model is, that the family *Syntrophomonadaceae* is mainly responsible for the conversion of butyrate to acetate via secondary fermentation. The produced acetate is mainly used by members of the *Methanosarcinaceae* but is also a main substrate for members belonging to the *Geobacteraceae*.

### Inhibition of methanogenesis

A further experiment with vegetable waste was conducted in the BES to reveal whether indeed methanogenesis can be accounted as the major reason for the loss of electrons in the reactors. 2-Bromoethanesulfonate is known as common inhibitor for methanogenesis as it represents a structural analogue of coenzyme M, which is required for methyl transfer [60]. Previous experiments revealed, that inhibition of methanogenesis by 2-bromoethanesulfonate could increase the coulombic efficiency from 35 up to 70%, even at low inhibitor concentrations of 0.27 mM [61].

**Fig. 5:**
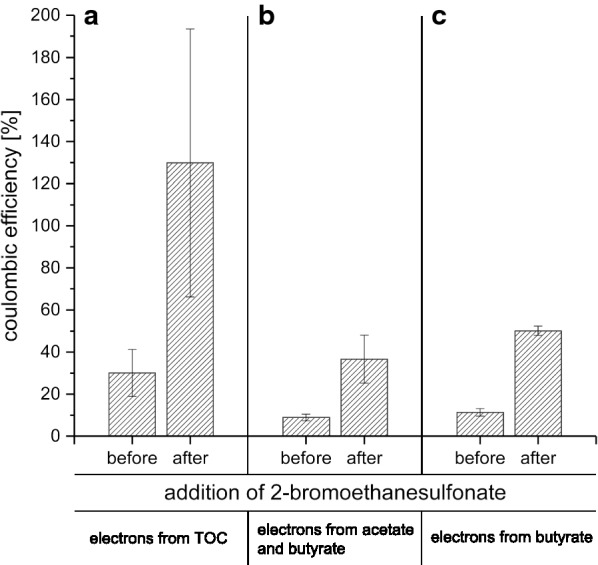
Calculated coulombic efficiency of the BES before and after the addition of 2-bromoethanesulfonate. The coulombic efficiency was calculated from the integral of the current curve and the quantity of electrons available from the amount of the degraded total organic carbon in the liquid phase (A) and from the amount of the degraded acetate and butyrate (B) or only butyrate (C)

Hence, the BES were started without 2-bromoethanesulfonate and ran for 7 days. Thereafter 2-bromoethanesulfonate was added to a final concentration of 50 mM. Concentrations of organic acids as well as TOC were determined before and after addition of 2-bromoethanesulfonate. With these values, coulombic efficiencies were calculated and can be extracted from Fig. 5.

As expected, the coulombic efficiency increased 4 to 4.5-fold after addition of bromoethanesulfonate, which supports the hypothesis that methanogenesis is the major competing factor in the BES. Consequently, it was also observed that the concentration of acetate as well as butyrate was more stable compared to the inhibitor-free control systems, where both acids were nearly completely oxidized. In the inhibitor-containing system, only 20% of the available acetate was degraded, while butyrate degradation was less restricted (72.7% of the available butyrate was degraded).

### Nanofiltration

Propionate was the major remaining end-product of the anode-assisted anoxic conversions. Propionate itself was presented as valuable platform chemical and could be used by other organisms as substrate for biotechnological reactions that could lead to more valuable end-products [62]. As propionate is metabolized via pyruvate, platform chemicals with metabolic pathways branching off at this intermediate should be especially suited for propionate based consumption.

First it was tested whether a higher concentration of propionate in the effluent of the BES could be achieved using nanofiltration membranes. For this purpose, the performance of two different membranes was compared in a stirring cell (see Table 4). Centrifuged and pre-filtered fermentate was concentrated with these membranes under nitrogen pressure. The NF90 membrane is supposed to have a tighter pore structure compared to the NF270 membrane. This could be corroborated by the filtration time, as the filtration process took about four to five times longer with NF90 compared to the NF270 membrane. Nevertheless, the better filtration result was achieved with the NF270 membrane, as the concentration factor for propionate was approx. 14.5% higher (see Table 4) and almost no propionate was found in the permeate.

**Table 4:** Conditions used for the nanofiltration process and the calculated concentration factor

Membrane type	Volume (ml)		Propionate (mM)		Concentration factor
	Start	End	Start	End	
Filmtec™ NF270	200	100	7.7	15.3	1.99
Filmtec™ NF90	200	100	10.5	17.8	1.70

### Production of platform chemicals

**Fig. 6:**
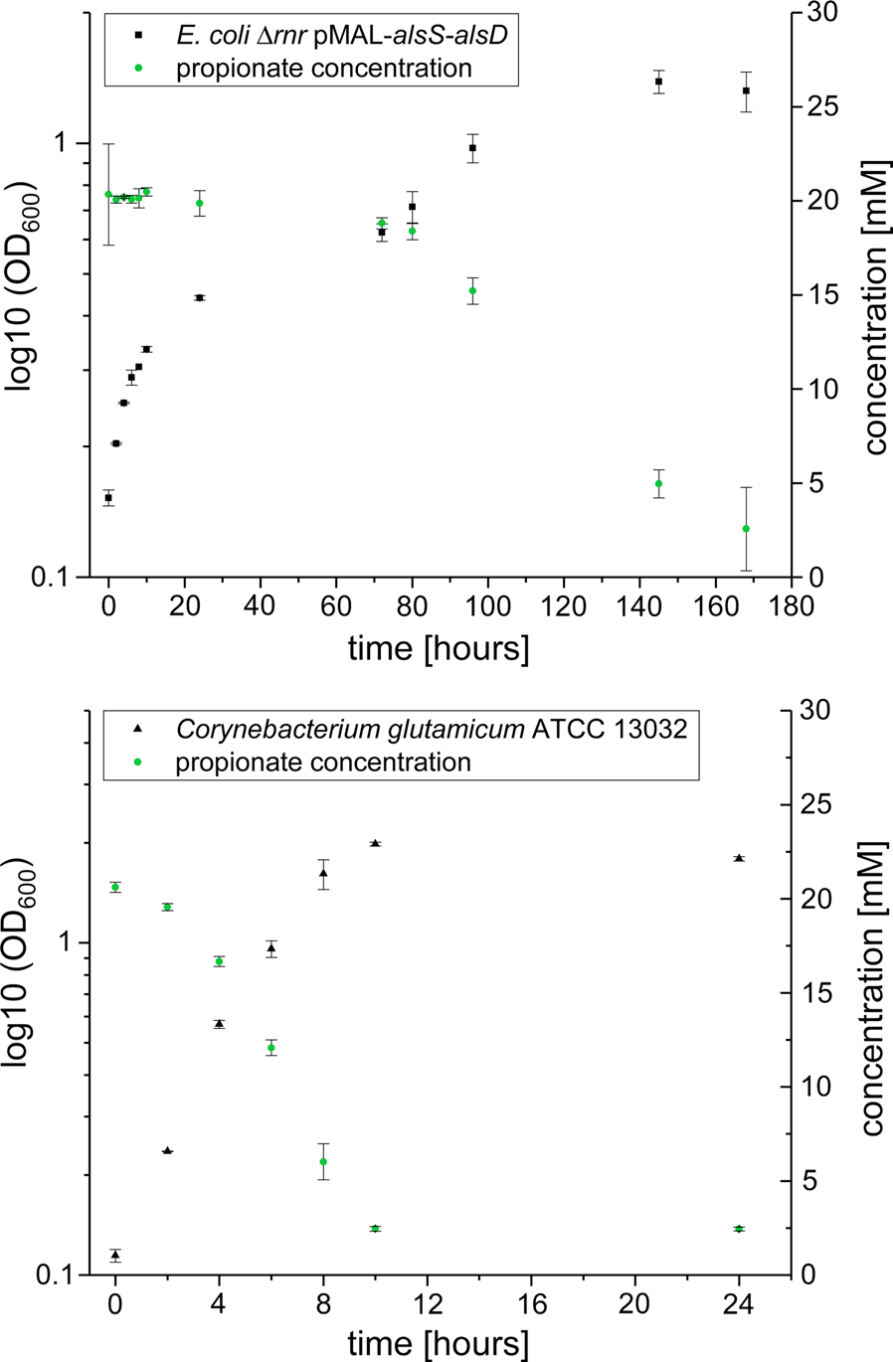
Growth experiments with *E. coli* Δ*rnr* pMAL-*alsS*-*alsD* (top) and *Corynebacterium glutamicum* (bottom). OD_600_ is shown in semi-log mode over time in hours. Fermentate from the BES without prior 2-bromoethanesulfonate addition, centrifuged and filtered with the crossflow system, was used as medium for both strains and pH fluctuations were prevented by addition of HEPES (50 mM). Both growth curves were conducted under oxic conditions. Propionate concentrations are given in mM, measured at same time points as OD_600_

In a first set of experiments, we tested whether the propionate containing effluent from the BES could be used as substrate for potential biotechnological production organisms. Hence, *C. glutamicum* and an *E. coli* strain were used for simple growth experiments (see Fig. 6). The *E. coli* strain contained a deletion of the *rnr* gene, which was previously presented to accelerate propionate consumption, especially under anoxic conditions [20]. As depicted in Fig. 6, both organisms grew under oxic conditions with the fermentate as only carbon and electron source. The addition of further supplements was not necessary. The doubling time for the *E. coli* strain was rather slow with 6.5 h, while *C. glutamicum* grew distinctly faster with a doubling time of 2.1 h. Also, *C. glutamicum* consumed the contained propionate distinctly faster than the *E. coli* strain.

As it could be shown that both strains are able to grow in the filtered fermentate and metabolize the contained propionate, it was of interest if these strains could produce any interesting chemicals out of the propionate.

Unfortunately, cell suspension assays with *C. glutamicum* lead to no detectable amounts of glutamate in the supernatant. Further examination will be necessary to investigate production of glutamate from propionate in *C. glutamicum.* Also, Penicillin G addition should be further characterized in terms of appropriate concentration, incubation time and OD_600_ at induction time point.

The cell suspension assay was also chosen to examine whether it could be possible to produce acetoin from the propionate of the fermentate. Therefore, the *E. coli* Δ*rnr* strain containing a plasmid for the heterologous expression of the *alsSD* genes (catalyzing the two metabolic steps from pyruvate to acetoin) was used. The cells were prepared as described, and the OD_600_ was adjusted to 2.5. At the end of the experiment, at 24 h, 3.12 mM of acetoin could be measured in the fermentate. Overall, 7.25 mM propionate were consumed in this timeframe. This ratio corresponds to a conversion rate of 86% of the theoretical maximum.

## Conclusion

This study reveals that vegetable waste can be a suitable substrate for the biotechnological production of platform chemicals. By the employed methods, namely dark fermentation combined with a bioelectrochemical system, a nanofiltration and, as last step, a biotechnological conversion, vegetable wastes could be converted to valuable platform chemicals and electrical energy. Our intention was to use the different organic acids from the hydrolysis step according to their biotechnological potential. Butyrate is metabolized via acetate and then fed into the citric acid cycle. Hence, using acetate or butyrate for the production of compounds branching of from pyruvate does not seem to be an efficient strategy as it would involve the energy consuming reaction to pyruvate first. This step is usually used for anabolic purposes only. In contrary, the propionate metabolism has pyruvate as its end product. Therefore, we believe that the production of electrons from acetate and butyrate in a bioelectrochemical system and platform chemicals from propionate is the best way of efficient usage of percolates from the hydrolysis reactor. Currently, the efficiency of the bioelectrochemical conversion of butyrate and acetate to carbon dioxide and electrons is hampered by methanogenesis. Nevertheless, this seems to be a problem that can be tackled by the design of reactors with higher surface to volume ratios since the bioelectrochemical oxidation of organic acids is thermodynamically more favorable compared to methanogensis. In other words, the currently used reactors provide a niche for methanogens by their high volume and low anode surface area, that can be omitted by the design of new reactors that favor productive biofilms. Our proof of principle experiments revealed the suitability of BES effluents as medium for biotechnological conversion or upcycling of propionate. Future steps will entail the development of production strains that are characterized by faster conversion rates.

## Additional file

**Additional file 1: Table S1.** Primer sequences and amplicon size for the 16S Illumina MiSeq.
