# Supplementary material for: Development of a production chain from vegetable biowaste to platform chemicals
Source: Microb Cell Fact. 2018 Jun 13;17:90. doi: 10.1186/s12934-018-0937-4 (PMC6001048; doi:10.1186/s12934-018-0937-4)
Supplement: Supplementary file 1 — Additional file 1: Table S1. Primer sequences and amplicon size for the 16S Illumina MiSeq. [file 12934_2018_937_MOESM1_ESM.docx]

Table S1. Primer sequences and amplicon size for the 16S Illumina MiSeq.

| **Primer** |  | **Primer sequence** | **Amplicon size** | **Reference** |
| --- | --- | --- | --- | --- |
| Bakt_341F | forward | CCTACGGGNGGCWGCAG | 464 bp | Klindworth et al., 2013* |
| Bakt_805R | reverse | GACTACHVGGGTATCTAATCC |  |  |
| A519F | forward | CAGCMGCCGCGGTAA | 387 bp |  |
| A906R | reverse | CAATTCMTTTAAGTTTC |  |  |

*Klindworth A, Pruesse E, Schweer T, Peplies J, Quast C, Horn M, et al. Evaluation of general 16S ribosomal RNA gene PCR primers for classical and next-generation sequencing-based diversity studies. Nucleic Acids Res. 2013;41:e1.
